# Decentralising the Self – Ethical Considerations in Utilizing Decentralised Web Technology for Direct Brain Interfaces

**DOI:** 10.1007/s11948-024-00492-2

**Published:** 2024-07-16

**Authors:** David M Lyreskog, Hazem Zohny, Sebastian Porsdam Mann, Ilina Singh, Julian Savulescu

**Affiliations:** 1 Department of Psychiatry, Warneford Hospital, University of Oxford, Warneford Ln, Oxford, OX3 7JX UK; 2 Wellcome Centre for Ethics and Humanities, University of Oxford, Oxford, UK; 3 Oxford Uehiro Centre for Practical Ethics, University of Oxford, Oxford, UK; 4 Faculty of Law, University of Oxford, Oxford, UK; 5 Centre for Biomedical Ethics, Yong Loo Lin School of Medicine, National University of Singapore, Queenstown, Singapore; 6 Murdoch Children’s Research Institute, Melbourne, Australia; 7 University of Melbourne, Melbourne, Australia

**Keywords:** Brain-Computer interfaces, Brain-Brain interfaces, Web3, Blockchain, Hybrid intelligence

## Abstract

The rapidly advancing field of brain-computer (BCI) and brain-to-brain interfaces (BBI) is stimulating interest across various sectors including medicine, entertainment, research, and military. The developers of large-scale brain-computer networks, sometimes dubbed ‘Mindplexes’ or ‘Cloudminds’, aim to enhance cognitive functions by distributing them across expansive networks. A key technical challenge is the efficient transmission and storage of information. One proposed solution is employing blockchain technology over Web 3.0 to create decentralised cognitive entities. This paper explores the potential of a decentralised web for coordinating large brain-computer constellations, and its associated benefits, focusing in particular on the conceptual and ethical challenges this innovation may pose pertaining to (1) Identity, (2) Sovereignty (encompassing Autonomy, Authenticity, and Ownership), (3) Responsibility and Accountability, and (4) Privacy, Safety, and Security. We suggest that while a decentralised web can address some concerns and mitigate certain risks, underlying ethical issues persist. Fundamental questions about entity definition within these networks, the distinctions between individuals and collectives, and responsibility distribution within and between networks, demand further exploration.

## Introduction

Technologies for Collective Minds (TCMs) are “technologies which facilitate and/or enable networks of two or more individuals who are sensing, thinking, and/or making decisions jointly in real time” (Lyreskog et al., 2023). These include brain-computer interfaces (BCI) and brain-to-brain interfaces (BBI), as well as alternative combinations of brain- and computer interfaces which may be referred to as brain-computer-brain interfaces (BCBIs) where the computer component, such as an artificial intelligence (AI) essentially functions as a co-thinker. These types of emerging technologies and constellations are not only potentially socially disruptive, but may also generate novel and substantial ethical challenges (Lyreskog et al., 2023; Danaher & Petersen, 2021; Hildt, 2015, 2019; Trimper et al., 2014). While some of these ethical issues may be taken to be intrinsic, for instance responsibility distribution or identity disruption, other issues such as privacy, transparency, safety and security may be to some degree contingent on the design of emerging and future TCM platforms. Some have argued that new decentralised web infrastructures, including cloud services and blockchains (Web 3.0, or simply ‘Web3’), could provide good bases for distributed and collective thinking technologies (Martins et al., 2019; Swan, 2016).

Consider a scenario where a network of people collaboratively discovers a cost-efficient alternative to penicillin through an online citizen science platform. In a conventional internet platform (Web 2.0), the data ownership, storage, access, and security are typically under the control of large tech corporations. This could lead to data misuse, vulnerability, or user-access denial. In contrast, a decentralised platform could grant data ownership to the participants and enhance data security by making the entries tamper-proof.

Ethically sound analyses of emerging TCMs need to take into consideration: (A) the technological specifications of any TCM; (B) the domain in which the TCM is deployed (military, medicine, research, entertainment, etc.); (C) reversibility. In this paper, we first, in Sect. ‘TCMs & WEB 3.0’, introduce TCMs and their potential in various domains, including military, medicine, and research. The realisation of this potential is, however, contingent *inter alia* on technical solutions to the problem of how TCM data should be transferred, processed, and stored. We also introduce blockchain and Web 3.0 technologies as a potential solution to this issue.

In the section  ‘The Ethics of Web 3.0 TCMs’, we analyse how decentralised TCMs may be able to handle the ethical issues and concepts normally pertinent to TCMs, in particular looking at (1) Identity, (2) Sovereignty (encompassing Autonomy, Authenticity, and Ownership), (3) Responsibility and Accountability, and (4) Privacy, Safety, and Security. We argue that while decentralisation may address some of these ethical issues, it also raises new concerns, particularly around the blurring of the sense of self and the distortion of concepts and values related to identity.

We conclude, in  ‘Conclusion: Decentralising the Self’, that despite the potential of blockchain and Web 3.0 technologies to alleviate some concerns and harms in TCM development and usage, fundamental uncertainties about entity definition, individual-collective distinctions, and responsibility distribution within TCM networks persist, complicating the ethical landscape. Concerns, largely stemming from a blurring of a sense of self^1^, continue to ripple through the domain, and to disrupt widely accepted moral frameworks for autonomy, privacy, and other core values. If anything, the decentralisation of properties and information we typically associate with a distinct and contained identity – such as thinking processes, and brain data – may lead to increased distortion of related concepts and values. Development of decentralised TCM systems will need to be sensitive to both older and novel ethical challenges in the design, build, and use. At this relatively early stage, research and development should be adapted accordingly to acknowledge and tackle substantial concerns in order to develop more ethical TCM systems, and to facilitate public acceptability.

## TCMs & WEB 3.0

TCMs come in many forms. While BCIs aim to improve and facilitate collective thinking by connecting human (or animal) brains to computers, BBIs offer another level of neural integration: direct brain-to-brain communication. These tools could be used for clinical, security, academic and social purposes, including direct communication with patients suffering from locked-in syndrome; coordination of military operations; gaming, or collaborative research. To deal with complexity, progress is being made on hybrid networks supported by artificial intelligence (AI) to coordinate and direct neural signals. These systems have mainly been tested for neuroprosthetics but are also being developed for improving communication between biological brains (Fares et al., 2022; Zhang et al., 2020; Jiang et al, 2019).

One of the challenges of TCMs – technologically and ethically – is the construction of a basis, or a platform, onto which interfaces are built, and over which data is sent, received, processed and/or stored. Two approaches have been tried. The first, applied mainly in BCIs, is closed loop offline systems. These systems are electronically supported by hardware and use software that does not require access to the internet to function. Deep Brain Stimulation (DBS) is an example of a closed loop BCI system. DBS can be calibrated and turned on or off either in a clinical visit or using remote-control operations. The system collects information that can then be transferred to a computer for storage and analysis, but there is no requirement for an Internet connection to perform these tasks.

The second method is to utilise the internet to coordinate and perform tasks through a brain interface. This approach has been used in BBI experiments, particularly where there are more than two participants, and/or where participants are geographically distant (Jiang et al., 2019; Stocco et al., 2015; Ramakrishnan et al., 2015). Such BBI experiments rely on the Internet as we know it today, sometimes referred to as “Web 2.0”. Where Web 1.0 presented users with static access to read-only content, Web 2.0 brought about the current participatory digital space allowing for effective, efficient, and multidirectional transfer of vast amounts of information. Though Web 2.0 has given rise to modern era infrastructures that rely on these informational capabilities for social, economic, and public health functions, among them BCI and BBI networks, these are subject to vulnerabilities stemming in part from their centralised nature. Most Web 2.0 data is stored centrally, in large server halls, by a handful of technology companies who own both servers and data. For TCM networks, in particular BBIs and BCBIs, this means that neural data, directly transmitted from people’s brains, may be stored and processed in specific geographical locations, and this data may be owned by large tech companies to varying degrees.

The issue of centralisation is among the primary motivations for the ongoing development of Web 3.0, the key feature of which is the use of blockchain and associated technologies to create decentralised online infrastructure for *inter alia* payments, data exchange and access control, and identity management. Blockchain technology refers to a set of advances in cryptography and computer science which collectively enable information to be distributed in near-real time to some or all members (‘nodes’) of a network in a secure and transparent manner (Haber & Stornetta, 1991; Nakamoto, 2008). Blockchains establish consensus among network participants on information exchanged through mechanisms that leverage economic or reputational incentives in a way that makes tampering with information contents prohibitively expensive (Lashkari & Musilek, 2021). Each node in a blockchain network is associated with one or more pairs of public and private keys. These keys are analogous to a username and password, allowing for identification and authentication, respectively. When combined with additional encryption software and personal data, these keys can be used to create decentralised identifiers (DIDs), which can be used as a privacy-protective method of identification and authentication.

DIDs are created and managed by the individual, allow for selective disclosure of associated personal information, and do not depend on third parties for management, storage, or sharing of data. As DIDs form part of the information that is shared between nodes in a blockchain network, the technology ensures that: (1) a network user cannot be blocked from accessing their own data; (2) the data cannot be tampered with; (3) the data can be accessed by the network user even if (multiple) servers storing the data are shut down or destroyed; (4) the user can decide to share some, rather than all, associated data (Mühle et al., 2018). While Web 2.0 applications can in principle provide some related services, such as end-to-end encryption and back-up servers, they remain vulnerable with regard to points 1–4.

Some have argued that blockchain could provide a sound basis for emerging TCMs. Many of the reasons for this are technical, in the sense that blockchain technology could alleviate or solve complex practical problems presented to brain interfaces and other TCMs. Martins et al. (2019), for instance, argue for the prospects of a Brain/Cloud interface:

“[A] B/CI might serve as a personalised conduit, allowing persons to obtain direct, instantaneous access to virtually any facet of cumulative human knowledge. Other anticipated applications include myriad opportunities to improve education, intelligence, entertainment, traveling, and other interactive experiences” (Martins et al., 2019, p1).

Swan (2016) similarly argues that such connections could benefit in numerous ways from utilising blockchain technology to secure the BCI and BBI connections:

“By linking brains to the Internet, BCIs could allow individuals to be more highly connectable not just to communications networks but also to other minds, and thus could enable participation in new kinds of collective applications such as a cloudmind. A cloudmind (or crowdmind) is the concept of multiple individual minds (human or machine) joined together to pursue a collaborative goal such as problem solving, idea generation, creative expression, or entertainment. The prospect of cloudminds raises questions about individual versus collective personhood. Some of the necessary conditions for individuals to feel comfortable in joining a cloudmind include privacy, security, reversibility, and retention of personal identity. Blockchain technology might be employed to orchestrate the security, automation, coordination, and credit-assignation requirements of cloudmind collaborations.” (Swan, 2016, p. 60).

So, there are technological and practical reasons to combine these technologies, but also ethical ones. Swan (2015, p.16), Martins et al. (2019) and others (Angelica et al., 2021) highlight some of the potential benefits, such as improved collaboration, cognitive enhancement, and improved empathy, as goods which we could pursue through using brain interfaces. These authors also suggest that concerns about neuroprivacy, safety and security could be alleviated by the use of blockchain technology in this domain. In what follows, we will anticipate and analyse the main ethical issues in using a decentralised web network to connect TCMs, with blockchain-based Web 3.0 solutions as a case illustration, while leaving open the possibility of other decentralised web solutions.

## The Ethics of Web 3.0 TCMs

We have previously called for more attention to be directed towards conceptual and ethical issues arising from emerging TCMs (Lyreskog et al., 2023). While there is a growing literature on the ethics of BCI and BBI technology, highlighting issues such as autonomy, privacy, and responsibility (Hildt, 2015, 2019; Trimper et al., 2014), we argue that new conceptual frameworks need to be developed to accommodate the novel entities which will be brought about by TCM networks, and that it is crucial to take into account the type of relations constituting the network as well as technical specifics. We consider a Web 3.0 platform which connects multiple agents through a direct brain interface^2^ and raises several ethical issues.

It is worth acknowledging that privacy, transparency, security, and safety issues related to TCMs are governed by various legal frameworks, such as the EU’s GDPR which regulates many aspects of data privacy. However, the primary focus of this paper is on the ethical dimensions and implications of these issues, which often extend beyond the scope of current legal frameworks and warrant philosophical analysis in their own right.

### Identity

While we usually think of ourselves as clearly connected to a private mental sphere, TCMs open up the possibility of a more fluid and potentially opaque sense of self – be it due to the distribution, alienation, abstraction, or opacity of our psychological or cognitive resources. In order to analyse how (other) moral values may be at stake, we need to be able to discern for whom those values may be at stake. Simply put: if multiple people share the same (albeit artificial) neuronal network, it may be unclear who exactly is contributing to which mental content and processing (Lyreskog et al., 2023). Let us consider an example.

#### CliMind

Imagine a group of researchers studying a complex phenomenon – say, climate change – using a TCM system. The researchers are connected through BCIs that in turn are connected to an artificial intelligence (AI) system. The AI system is designed to analyse their individual neural data – representing their thoughts, ideas, and analyses – and to coordinate that data into a collective, coherent interpretation of the climate data the researchers are reviewing.

Here, the researchers’ sense of self may be disrupted as thoughts and ideas are pooled and processed collectively, making it difficult to discern who contributed what. It might be that a novel insight into climate change emerges from this process, but no single person can claim it as their own – it is truly the product of their (and the AI’s) collective cognition.

However, using Web 3.0 technology, each researcher’s contributions could be marked by unique but anonymisable DIDs^3^. This would allow tracking of the origin of specific neural data and securely linking them back to their source (the individual researcher). In this way, each researcher maintains a unique mark of identity within the collective, rather than having their contributions absorbed anonymously into the group cognition.

In this way, a decentralised web may provide a more ethically sound base for TCM systems, allowing participants to maintain ownership and sense of self, instead of these being obscured or contingent on the transparency of large tech corporations. This, indeed, is one of the reasons why decentralised web applications may be better suited than Web 2.0 to host TCM networks, from an ethical perspective: identity can be owned and instantiated by each network participant, rather than by corporations owning distinct platforms. However, there are still issues that need unpacking in this domain.

Even with participant identifiers, and with tracking of their cognitive contributions, it remains unclear how we should best understand representations of individuality in a network like CliMind, particularly in cases where the network results in emergent phenomena which cannot be fully explained by individual contributions: is it (CliMind) “merely” a collective, or is it better viewed an entity in its own right? If the latter, then when becomes of the individual participants?^4^

Addressing the disruptive power of this technology in the domain of identity, Swan (2016) proposes we let go of the historically important concept of ourselves as singular and coherent entities over time, and instead embrace a self-understanding which allows multiple versions and aspects of us – a “identity multiplicity”:

“In the case of BCI cloudminds, identity multiplicity might involve many different forms of participation. There could be “classic meatspace brains,” one or more digital selves, and different configurations of selves[…]. Human beings are currently constrained to an embodied form; however, this may not be the situation in the future. Digital identities might become so distributed, portable, easily copied, open-sourceable, sharable, and malleable that it no longer makes sense to think in terms of distinct entities but rather in some other parameter such as instances.” (Swan, 2016, p74).

Prima facie, this approach to dealing with concerns about identity in decentralised TCM networks is attractive. Indeed, widely adopted frameworks for thinking about agency struggle to deal with the challenges posed by emerging TCMs, as their criteria for both individual and collective agency map poorly onto TCM networks – partly due to unclear individuation protocols: it is not clear what in a network constitutes a (separate, unique) entity, and what does not (Lyreskog et al., 2023). However, the definition of “identity” in this context is somewhat vague. Swan (2016), for instance, does not make it clear to what extent we should interpret the concept of “identity multiplicity” as a metaphysical state, or merely as instantiations of self-expression (or something in-between). If the former interpretation is the most apt, we would need to adopt or develop some form of account for what such a metaphysical state might look like, e.g. a parallel psychological continua (Parfit, 1984) or perhaps some form of interpersonal extended mind theory (León et al., 2019). Additionally, arguing that we ought to retire the concept of a (single) solid self, or identity overall, is all well and good until we need to anticipate and shape alternatives. Resorting to concepts of collectivity and joint action will plausibly not suffice, as the problem of identity ascription remains in at least two ways. First, we may ask what is the identity of such a collective? And what is a collective, if not a collection of individuals? Second, if we accept the idea that identity in itself will be splintered – or at the very least fundamentally re-configured – (other) moral values tied to it will, too, be correspondingly splintered or otherwise augmented. This central problem – the confounding conceptual implications of decentralising identity, and, thereby, potentially our very selves – as we shall see below, ripples through the moral landscape of TCMs. Consequently, the problem does not only remain unsolved by using decentralised technologies as a basis for TCM networks, but could potentially worsen it.

### Sovereignty: Autonomy, Authenticity, and Ownership

One of the key possibilities brought about by the decentralised web is to allow individuals participating in a given network the freedom to act according to their own values and preferences, with little to no interference by centralised governance. For instance, a “smart contract” (a central function for Web 3.0 transactions) is a self-executing program that automatically enforces the terms of an agreement between parties, so that no mediator or executor is required (Szabo, 1994; Buterin, 2014). In the context of emerging TCMs, this is preferable to current Internet networks, as the latter may directly intervene in, and control neural data flow. This is, however, problematic for a number of reasons.

Some have raised concerns about the possibility of direct TCMs leading to mind control scenarios, where singular or few agents control the thought processes and neural data of many, limiting freedom of thought and (therefore) personal autonomy (Ienca & Haselager, 2016; Hongladarom, 2015). For instance, we can imagine a scenario where a TCM protocol is written so that some network participants have more sway than others, making their personal preferences count for more within the network – and this uneven power structure could be completely opaque to the (other) participants. Relatedly, if such a scenario were to occur, questions would arise about to what extent our individual thoughts and ideas were truly our own. This is partly a moral-philosophical issue pertaining to the value of authenticity: are my ideas expressed in a TCM network truly mine, if someone is directly manipulating and perhaps censoring my very ability to conjure that idea? It is also in part a legal problem of ownership: what are the implications for copyright law and frameworks for intellectual property, if centralised (presumably for-profit) entities enable, hold, and legally own the neural data producing the idea?

A decentralised web may mitigate some of these concerns about the sovereignty of our thoughts and minds^5^. The autonomy of participants’ thoughts and ideas could be supported by ensuring that no one entity has control over neural data traffic, and that the origins of (contributions to) those thoughts and ideas are accurately and safely traceable back to their original identifiers, in their original formulation, thereby mitigating uncertainties regarding ownership.

However, these mitigating factors are only solutions insofar as they address these concerns based on the assumption that we can and should make sense of individuation in a traditional sense. As we saw in the previous section, it is far from clear that we should conceptualise agency and identity in this way. As a consequence, it is equally unclear to what extent decentralisation affects sovereignty, as questions will linger about *whose* autonomy and ownership over ideas and thoughts we should be concerned with, and what might constitute their authentic, true self (if a TCM network can reasonably be said to have such a thing). Furthermore, assuming TCMs can and will give rise to emergent phenomena which amounts to more than the sum of each individual contribution to a TCM network, the traceability of neural data will not only depend on our ability to trace (parts of) ideas back to their original sources, but to identify to what extent those sources, respectively, have contributed to the emergent phenomena. This task will be difficult, not to say impossible, even on a Web3 platform.

Additionally, the geographical aspects of decentralisation put stress on the common inclination to locate sovereignty within a specific, physical place. In the same way we can point to a (albeit somewhat general) place and time at which the French Revolution took place, we tend to think that we can locate our thoughts, ideas, and decisions at some (perhaps equally general) place in space-time. Take for instance the following sentence:

When I woke up in my bed this morning, I decided to quit my job.

In this case, we pinpoint where and when a certain mental action was performed, and quite comfortably so: the thought is to quit one’s job, the source of the thought is me – my mind, and/or organism, located in bed – this morning. In a TCM setting – perhaps a BBI network – our intuitions may be somewhat more unclear, as who exactly is involved in the (co-)thinking, where, and when, is not certain. Using a decentralised platform risks further obfuscating the time and location, and identifying which thoughts are generated, as the process is purposefully distributed throughout the network, so that the data traffic is processed and held at multiple locations. Additional complexity is added by the tendency of thoughts, ideas, and decisions to often be unstructured – or at the very least less structured than our example sentence above – and in themselves distributed and formulated over time^6^. Not only may this alienate us from thought processes in which we take part – as they may be less seen as truly our own – but it potentially has major consequences for how we conceive of what it means to be responsible for those thoughts, and subsequently to what extent anyone can be said to be accountable for them.

### Responsibility and Accountability

One of the larger ethical challenges with TCM networks concerns the accurate ascription of responsibility and accountability for outcomes brought about by a given network. The term ‘responsibility’ is contentious, with numerous competing accounts (Vargas, 2013; Talbert, 2016; Bivins, 2006; Fischer & Ravizza, 1998). For our purposes, it can be broadly understood as denoting the relationship between a moral agent and outcomes of actions which are in some (morally) relevant way linked to that agent. E.g., a moral agent A is responsible for a (morally relevant) event E iff A knowingly caused E understanding what it could reasonably entail. Accountability, while closely related to responsibility, is not synonymous. Although definitions vary in the literature, a simple (and for our purposes sufficient) description is that a person “can be held accountable if (1) the person is functionally and/or morally responsible for an action, (2) some harm occurred due to that action, and (3) the responsible person had no legitimate excuse for the action.” (Bivins, 2006, p.21).

One could argue that we ought to move away from evaluating morally charged phenomena in terms of binary constructs (i.e. either something is individual, or it is collective – be it agency, responsibility, or praiseworthiness), and begin assessing them based on other criteria. One such criterium could be the capacity for moral deliberation and decision-making. Normally we do not, for instance, assess the moral culpability of animals or small children based on whether there are many or few of them, but based on their capacity to understand and act on morally salient information – regardless of whether that capacity is held by an individual or a collective. As an entity gains more understanding of morally salient information, greater ability to act upon it, and so forth, we tend to gradually ascribe moral responsibility to a higher and higher degree. However, we would seemingly still want to say that “this or that entity” has moral responsibility for X, in which case we would need to individuate that entity in a fair way, and we are back to square one: what constitutes an entity in a TCM network?

Similarly, accountability for actions or ideas generated by TCM networks can be a tricky matter, depending on a couple of parameters. The first parameter is purely technical: if a decentralised TCM network utilises anonymous (or anonymisable) DIDs, it will be impossible to link an individual’s contribution to an action to their real-world persona – which presumably is the person we wish to hold accountable.^7^ The specific DID may perhaps suffer some reputational damage depending on what they have caused through the action, but only insofar as the networks to which they are connected care about the action.^8^ If, on the other hand, DIDs are linked to real-world persons, accountability could presumably more readily located.

The second parameter is the appropriateness of accountability ascription over time. Let us revisit the hypothetical case used earlier: a TCM network of people using an online citizen science platform to research infectious disease, and as a result discovering an effective and cost-efficient alternative to penicillin. Now let’s imagine that cognitive process(es) leading to this discovery continue to work, and lead to the discovery of a bioweapon. If the network requires DIDs, the sources of the discovery can be traced to specific participant DIDs. Furthermore, if those DIDs can be traced to specific persons, we could trace participation down to who participated with what processes and when. This could be helpful in ascribing accountability, as we for instance may be able to see if anyone *only* contributed to the penicillin, and dropped out before the processes led to the discovery of a bioweapon. However, it may not be clear that all persons participating in the network at the time of the discovery of the weapon (a) knew what they were contributing to, or (b) tried to contribute to it, and/or (c) contributed substantially to it. This obfuscates accountability ascription in decentralised contexts, as it will be unclear who should be held accountable for what – even if all participators are traced with de-anonymised DIDs.

### Privacy, Safety & Security

One of the key possibilities of the decentralised web is encryption-protected anonymity. This allows users strict control over how their data is shared and used: if I do not want to share my data, nobody can demand it or take it from me – partly due to the security of encrypted identifiers (be they public keys or DIDs) making access difficult, and partly due to the fact that they simply do not know who I am. The decentralised nature of DIDs could furthermore add security, as the data will be protected by a distributed security protocol, making tampering with the data all but impossible. In the context of TCM networks, this could be a major advantage in terms of providing users with privacy, security, and safety of personal data, including neurodata. However, this is assuming that the privacy, security, and safety of a TCM network – of a collective mind – should be interpreted in terms of the equivalent values of its individual constituents, that is that a TCM network provides adequate privacy, safety, and security, if and only if the participants of that network enjoy adequate privacy, safety, and security, from an individual perspective.

It is unclear, however, to what extent we should conceptualise privacy, safety, and security in this way when assessing the ethical impacts of TCMs. Arguably, the very idea of neural privacy seems closely related to the notion of mental integrity at an individual level (Zohny et al., 2023). If neural information is shared, it is no longer private in the same sense – the sense in which we may believe people have a right to privacy – but perhaps more so the sense in which a social event, or a conversation in confidence between two good friends, can be private. Were I to share my cognitive/neural network with another person through a TCM, it may seem odd to claim a right to privacy of *my* thoughts, as distinct from those of the other. If so, it could be that the right to privacy, and its related concepts, need to be completely reformulated, and it is unclear to what extent a decentralised web will provide adequate protection under those new definitions (Zohny et al., 2023).

## Conclusion: Decentralising the Self

Technologies for Collective Minds (TCMs) raise a number of ethical issues. Some have argued that these issues can be mitigated by using a decentralised Internet (Web 3.0) as a basis for TCM networks. In this paper, we have analysed these claims, and shown how decentralisation could affect key ethical values in the TCM domain, in particular issues about (1) Identity, (2) Sovereignty (understood as a compound of Autonomy, Authenticity, and Ownership), (3) Responsibility and Accountability, and (4) Privacy, Safety, and Security. We argue that, while a decentralised web can address some of these concerns, and mitigate potential harms in TCM development and usage, underlying issues remain. Fundamental questions about what constitutes an entity in TCM networks; what (if any) the relevant differences between individuals and collectives are; and how to conceive of responsibility distribution within TCM networks, continue to muddle the waters.

As technology development progresses, widely adopted concepts may become obsolete, or insufficient for purposes of ethical analysis. Concepts such as identity, sovereignty, responsibility, and the like will likely require revision in the light of developments in brain-computer and brain-brain interfaces. Research ought to be pursued across domains, to better understand how we (should) value and understand key elements of emerging technologies for collective thinking and decision-making: conceptual analysis of the nature of agency, identity, and responsibility of TCM networks; empirical work into how people *de facto* perceive and value participation in TCM networks; normative analysis of how key values are best pursued in R&D, policy, and praxis. These investigations should remain flexible, and sensitive to: (a) relevant technological specifications of TCM applications; (b) the specific domain for which those applications are being developed and/or employed into; (c) the reversibility of the applications.

## Footnotes

1 The term ‘sense of self’ here is intended as a phenomenological descriptor, broadly encompassing feelings and experiences pertaining to identity retention. We take this to be separate from the idea of *de facto* identity retention, which, while intimately intertwined to our sense(s) of self, in turn can denote any number of phenomena, including (but not limited to) legal identity; psychological continuity; self-narrative coherence; social positioning, etc. As we take it, these can all in varying degree affect our sense of self, and, conversely, be affected by it. We here discuss the problem of identity in terms of ‘sense of self’ for practical reasons.

2 Lyreskog et al. (2023) define these entities as “multi-directional and direct”, or “MacroMinds”. These are typically constituted by human agents (and possibly artificial/hybrid agents) which are connected through a BBI or BCBI.

3 That is, the design of DIDs is such that the data stored in connection to them is not necessarily linked to any specific person or organisation, but *can* be if required. Any one person or organisation can in principle have multiple DIDs, for various purposes, where some may be publicly linked to them, while others may not be. (Sporny et al., 2021)

4 We have elaborated on this problem elsewhere (Zohny et al., 2023). Worth emphasising here, however, is that TCM networks are not likely to be homogenous in the way they operate: while some may quite easily lend themselves to analysis and tracking, other more complex “MacroMinds” may be more difficult to dissect fairly – in particular where they generate complex systems resulting in emergent phenomena.

5 For the purposes of the argument, we here tentatively use the term “sovereignty” to denote a conceptual compound containing the constituent concepts of autonomy, authenticity, and ownership. Although a deeper conceptual analysis of the interrelations between these concepts may be warranted, we leave this project for another time. For now, it suffices to say that there is no claim that these three concepts exhaust the concept of sovereignty – even in the specific domain at hand. They are, however, prevalent in the literature relevant to the same domain, under a variety of definitions.

6 The authors would like to thank an anonymous reviewer for this observation.

7 A case could be made to hold DID-based avatars (as opposed to the persons owning and controlling the DIDs) morally responsible, and by extension accountability could be more readily located. However, it’s not clear that this is a very attractive route, as we tend to want to hold only moral agents morally responsible. Unless the avatars display the capacities necessary for moral agency, holding them morally responsible may prove to be a challenge.

8 One could imagine a system aimed at tracking or facilitating accountability could be implemented using cryptocurrency tokens representing either economic or reputational capital.
